# Supplementary material for: A Critical View of the Application of the APEX Software (Aqueous Photochemistry of Environmentally-Occurring Xenobiotics) to Predict Photoreaction Kinetics in Surface Freshwaters
Source: Molecules. 2019 Dec 18;25(1):9. doi: 10.3390/molecules25010009 (PMC7017383; doi:10.3390/molecules25010009)
Supplement: Supplementary file 1 [file molecules-25-00009-s001.zip › 3.2.4_gcc-4.4.0.zip!/3.2.4_gcc-4.4.0/3.2.4_gcc-4.4.0/doc/octave/html/interpreter/System-Information.html]

System Information - Untitled


Next: Hashing Functions,
Previous: Group Database Functions,
Up: System Utilities


---

### 34.11 System Information

— Function File: [c, maxsize, endian] = **computer** ()  
> Print or return a string of the form cpu-vendor-os
> that identifies the kind of computer Octave is running on. If invoked
> with an output argument, the value is returned instead of printed. For
> example,
>
> ```
>           computer ()
>                -| i586-pc-linux-gnu
>           
>           x = computer ()
>                => x = "i586-pc-linux-gnu"
> ```
>
> If two output arguments are requested, also return the maximum number
> of elements for an array.
>
> If three output arguments are requested, also return the byte order
> of the current system as a character (`"B"` for big-endian or
> `"L"` for little-endian).

— Built-in Function: [uts, err, msg] = **uname** ()  
> Return system information in the structure. For example,
>
> ```
>           uname ()
>                => {
>                      sysname = x86_64
>                      nodename = segfault
>                      release = 2.6.15-1-amd64-k8-smp
>                      version = Linux
>                      machine = #2 SMP Thu Feb 23 04:57:49 UTC 2006
>                    }
> ```
>
> If successful, err is 0 and msg is an empty string.
> Otherwise, err is nonzero and msg contains a
> system-dependent error message.

— Function File: **ispc** ()  
> Return 1 if Octave is running on a Windows system and 0 otherwise.
>
>
>
> **See also:** ismac, isunix.

— Function File: **isunix** ()  
> Return 1 if Octave is running on a Unix-like system and 0 otherwise.
>
>
>
> **See also:** ismac, ispc.

— Function File: **ismac** ()  
> Return 1 if Octave is running on a Mac OS X system and 0 otherwise.
>
>
>
> **See also:** ispc, isunix.

— Built-in Function: **isieee** ()  
> Return 1 if your computer claims to conform to the IEEE standard for
> floating point calculations.

— Built-in Function: **OCTAVE\_HOME** ()  
> Return the name of the top-level Octave installation directory.

— Built-in Function: **OCTAVE\_VERSION** ()  
> Return the version number of Octave, as a string.

— Function File: **license**  
> Display the license of Octave.
> — Function File: **license** ("inuse")  
> > Display a list of packages currently being used.
> > — Function File: retval = **license** ("inuse")  
> > > Return a structure containing the fields `feature` and `user`.
> > > — Function File: retval = **license** ("test", feature)  
> > > > Return 1 if a license exists for the product identified by the string
> > > > feature and 0 otherwise. The argument feature is case
> > > > insensitive and only the first 27 characters are checked.
> > > > — Function File: **license** ("test", feature, toggle)  
> > > > > Enable or disable license testing for feature, depending on
> > > > > toggle, which may be one of:
> > > > >
> > > > > "enable": Future tests for the specified license of feature are conducted as usual. "disable": Future tests for the specified license of feature return 0.
> > > > >
> > > > > — Function File: retval = **license** ("checkout", feature)  
> > > > > > Check out a license for feature, returning 1 on success and 0
> > > > > > on failure.
> > > > > >
> > > > > > This function is provided for compatibility with matlab.
> > > > > >
> > > > > >
> > > > > >
> > > > > > **See also:** ver, version.

— Function File: **version** ()  
> Return Octave's version number as a string. This is also the value of
> the built-in variable `OCTAVE_VERSION`.

— Function File: **ver** ()  
> Display a header containing the current Octave version
> number, license string and operating system, followed by
> the installed package names, versions, and installation
> directories.
> — Function File: v = **ver** ()  
> > Return a vector of structures, respecting Octave and each installed package.
> > The structure includes the following fields.
> >
> > `Name`: Package name. `Version`: Version of the package. `Revision`: Revision of the package. `Date`: Date respecting the version/revision.
> >
> > — Function File: v = **ver** (`"Octave"`)  
> > > Return version information for Octave only..
> > > — Function File: v = **ver** (pkg)  
> > > > Return version information for the specified package pkg.
> > > >
> > > >
> > > >
> > > > **See also:** license, version.

— Built-in Function: **octave\_config\_info** (option)  
> Return a structure containing configuration and installation
> information for Octave.
>
> if option is a string, return the configuration information for the
> specified option.

— Loadable Function: **getrusage** ()  
> Return a structure containing a number of statistics about the current
> Octave process. Not all fields are available on all systems. If it is
> not possible to get CPU time statistics, the CPU time slots are set to
> zero. Other missing data are replaced by NaN. Here is a list of all
> the possible fields that can be present in the structure returned by
> `getrusage`:
>
> `idrss`: Unshared data size. `inblock`: Number of block input operations. `isrss`: Unshared stack size. `ixrss`: Shared memory size. `majflt`: Number of major page faults. `maxrss`: Maximum data size. `minflt`: Number of minor page faults. `msgrcv`: Number of messages received. `msgsnd`: Number of messages sent. `nivcsw`: Number of involuntary context switches. `nsignals`: Number of signals received. `nswap`: Number of swaps. `nvcsw`: Number of voluntary context switches. `oublock`: Number of block output operations. `stime`: A structure containing the system CPU time used. The structure has the elements `sec` (seconds) `usec` (microseconds). `utime`: A structure containing the user CPU time used. The structure has the elements `sec` (seconds) `usec` (microseconds).
